# Disseminated talaromycosis in an HIV-infected patient

**DOI:** 10.1590/0037-8682-0896-2020

**Published:** 2021-03-22

**Authors:** Chee Yik Chang, Adrena Abdul Wahid, Edmund Liang Chai Ong

**Affiliations:** 1 Hospital Sultanah Aminah, Department of General Medicine, Johor, Malaysia.; 2 Hospital Sultanah Aminah, Department of Pathology, Johor, Malaysia.; 3 University of Newcastle Medical School, Newcastle upon Tyne, United Kingdom.

**FIGURE 1:**
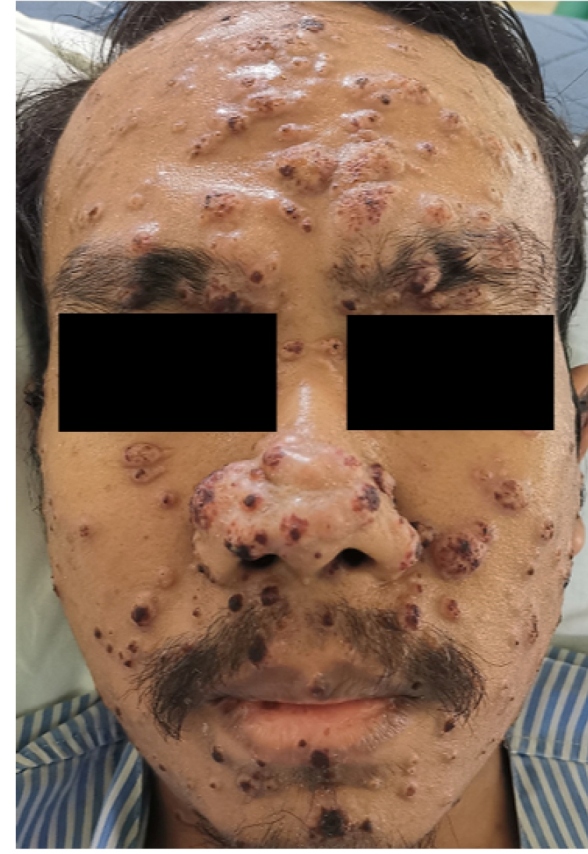
Multiple plaques and nodules with raised edges and central crust seen over the face.

**FIGURE 2:**
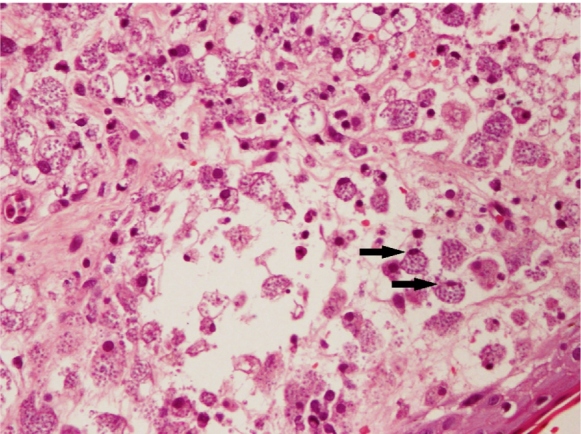
Histopathological examination of the skin biopsy specimen revealing fungal bodies within the macrophages (arrow).

A 25-year-old man with newly diagnosed human immunodeficiency virus (HIV) infection (CD4 count = 53 cells/mm^3^) presented with a one-month history of generalized cutaneous lesions starting at the trunk and spreading to the face and limbs. The patient experienced intermittent fever, fatigue, and weight loss, but no cough, breathlessness, abdominal pain, or diarrhea. Physical examination revealed multiple plaques and nodular lesions with raised edges and a central crust **(** Figure 1 **)**. Abdominal ultrasonography showed splenomegaly (spleen length=15.9 cm). Skin biopsy revealed abundant fungal spores highlighted by positive periodic acid-Schiff (PAS) and Grocott's methenamine silver (GMS) stains **(** Figure 2 **)**. His blood culture revealed growth of *Talaromyces marneffei*, and hyphae were visualized on Gram stain. The skin lesions improved following treatment with intravenous amphotericin B for two weeks, followed by oral itraconazole as consolidation and maintenance therapy. Antiretroviral therapy and antifungal therapy were initiated.

Talaromycosis is a deep fungal infection caused by *Talaromyces marneffei* endemic in Southeast Asia and the southern part of China^1^. HIV is a major risk factor for talaromycosis in endemic regions. Infection occurs predominantly in patients with advanced HIV disease, with a CD4 count below 100 cells/mm^3^ ^2^. Patients with talaromycosis most commonly present with fever, weight loss, and anemia. Skin lesions on the face, upper trunk, pinna, and arms are a characteristic of dissemination^1^. Cutaneous lesions, a useful diagnostic sign in talaromycosis, have a typical central-necrotic appearance. However, they are often a late sign of talaromycosis and can be absent in up to 60% of cases^3^. A review of 155 cases of talaromycosis revealed skin and blood as the most frequent sites of culture-positive infection with 96 (62%) and 85 (55%) cases, respectively, whereas the spleen was involved in eight cases (5%)^1^. Untreated disseminated talaromycosis is associated with fatality. Treatment includes induction therapy with amphotericin B, followed by consolidation and maintenance therapy with oral itraconazole^1^ ^,^ ^2^.
